# Psychiatric premises for abortion in Poland - ethical, legal and clinical issues

**DOI:** 10.1192/j.eurpsy.2024.1199

**Published:** 2024-08-27

**Authors:** W. E. Kosmowski

**Affiliations:** Department of Psychiatry, Nicolaus Copernicus University, Bydgoszcz, Poland

## Abstract

**Introduction:**

After judgment of the Constitutional Tribunal of 22.10.2020, there are two premises for abortion: when pregnancy was caused by rape or pregnancy is the threat for health and life of a mother. Then some people indicated that the latter should be interpreted more broadly. So far, jurisprudence has interpreted health threats only in relation to physical health, currently – cases classified as mental health threats are included.

**Objectives:**

The aim of this paper is first to analyze different aspects of this phenomenon: clinical, philosophical, including ethical and legal. The second goal is to point out the best actions for psychiatrists.

**Methods:**

The methodology of this paper corresponds to the pastoral paradigm: diagnosis, reflection, action. At first, the arguments of opponents and proponents of the concept of psychiatric premises for abortion were extracted. Then they were assessed from a logical and essential point of view. Finally, some conclusions and guides were included to enable psychiatrists to act appropriately, including ethical, clinical and legal aspects.

**Results:**

Statements and letters from various institutions and societies were analyzed, including the Presidium of the Supreme Medical Council, Polish Pediatrics Society, the Expert Team on Bioethics of the Polish Bishops’ Conference, the Bioethics Committee of the Polish Academy of Sciences, Patient’s Rights Ombudsman, Commissioner for Human Rights. The key arguments for psychiatric premises are presented in the Table 1.

**Table 1.** 

Argument	Description
Etiological	the only cause of mental disorders during pregnancy is the pregnancy itself or fetal diseases
Therapeutical	abortion is a method of treating mental disorders during pregnancy
Prognostic	possible long- and short-term complications after the abortion procedure do not pose a significant threat to the woman’s life and health
Consultation-Liaison	the task of the consultant psychiatrist is to indicate what actions other doctors should take
Ethical	the value of the fetus’s life is negligible compared to values such as the mother’s mental state or well-being
Political	such conduct is beneficial to state policy and the good of society
Legal	such procedures are legal

According to opponents, using the premise of mental health risks to terminate a pregnancy would be an example of the psychiatrization of life and the abuse of psychiatry for political purposes. There would be a danger of associating psychiatry as a tool for performing abortions, which would perpetuate the phenomenon of stigmatization – of both doctors and patients. Each of the arguments for this has been negated.

**Conclusions:**

This problem illustrates an attempt to replace the paradigm of traditional personalistic ethics with utilitarianism. The concept of psychiatric premises for abortion is contrary to the principles of double effect and proportionality. It is also against the Polish Code of Medical Ethics: art. 39 and art. 54.

**Disclosure of Interest:**

None Declared

